# Health status and mental distress in people with cancer and comorbid conditions: The Australian National Health Survey analysis

**DOI:** 10.1002/cam4.6291

**Published:** 2023-06-23

**Authors:** Huah Shin Ng, Richard Woodman, Bogda Koczwara

**Affiliations:** ^1^ Flinders Health and Medical Research Institute, College of Medicine and Public Health Flinders University Adelaide South Australia Australia; ^2^ SA Pharmacy Flinders Medical Centre Bedford Park South Australia Australia; ^3^ Department of Medical Oncology Flinders Medical Centre Adelaide South Australia Australia

**Keywords:** cancer management, epidemiology, risk assessment, statistical methods

## Abstract

**Introduction:**

Data on the impact of specific comorbidities on health outcomes is limited. We compared health status and mental distress between individuals with and without cancer according to comorbidity type.

**Methods:**

A cross‐sectional analysis using data from the Australian National Health Survey 2017–18 including all respondents aged ≥25 years with and without a history of cancer. The odds of poor health and mental distress were reported according to cancer status, and specific individual and cluster of comorbidities.

**Results:**

There were 1982 individuals (52% female) with cancer and 12,635 (51% female) without cancer. Individuals with cancer were older, and more likely to have a comorbidity compared with those without cancer. They were more likely to report poor health than those without cancer for each specific comorbidity; except for skin conditions and infectious diseases; with the adjusted odds ratio (aOR) ranging from 1.34 (95% CI = 1.01–1.79) for digestive disorders to 2.93 (95% CI = 1.62–5.29) for blood conditions. The strongest association with poor health (aOR 2.79, 95% CI = 2.27–3.43) and mental distress (aOR 9.01, 95% CI = 7.25–11.20) was observed for those with a comorbid mental illness. Exploratory cluster analysis identified four distinct comorbidity clusters: low comorbidity, musculoskeletal, respiratory and cardiometabolic; cancer survivors in the cardiometabolic cluster had a higher odds of reporting poor health (aOR 3.50, 95% CI = 2.48–4.92) and mental distress (aOR 2.33, 95% CI = 1.53–3.55) than those with a low comorbidity.

**Conclusions:**

Comorbidities in cancer survivors were common and associated with inferior health status, although the magnitude of the effect varied by comorbidity type. Risk assessment and management of comorbidities should be an important priority for cancer care and research.

## 1 INTRODUCTION

The coexistence of one or more chronic health conditions (‘comorbidities’) is common in people with cancer,^1^, ^2^ with over 60% of cancer survivors suffering from at least one comorbidity^3^, ^4^, ^5^ and the prevalence of comorbidities is higher in cancer survivors compared with the general population. This may be due to shared risk factors between cancer and other chronic diseases such as smoking, obesity, inactivity and poor diet.^2^ It is also possible that people with select chronic conditions are at higher risks of developing cancer such as the association between type 2 diabetes and an increased risk for several cancers including breast, colorectal, endometrial, pancreatic and liver cancer.^2^, ^6^ Enhanced monitoring and diagnostic testing following cancer diagnosis may also increase the incidental finding of comorbidities.^3^ Further, cancer treatment may contribute to the development of comorbidities, including cardiovascular diseases (associated with chemotherapy, immunotherapy, targeted therapy, endocrine therapy, or radiotherapy),^7^ and osteoporosis (associated with endocrine therapy or chemotherapy‐induced ovarian failure).^8^ In recognition to the accompanying risks of toxicity associated with cancer treatment, several clinical guidelines have been developed with suggested strategies for detection, prevention and management of cardiovascular complications and osteoporosis in cancer patients.^7^, ^8^

Comorbidity is associated with shorter survival and lower quality of life, with evidence suggesting worse outcomes in cancer survivors compared to the general population.^2^ While prior Australian studies showed that people with cancer had a greater number of chronic diseases, poorer health status and a higher level of distress than people without cancer,^1^, ^3^ data on the influence of specific comorbidities on health outcomes is limited. A greater understanding on the burden of individual comorbidity is needed to identify population most affected who may benefit the most from prevention and early intervention.

The objective of this study was to assess the patterns of health outcomes in individuals with cancer and comorbidities. We compared the prevalence and strength of the association between comorbidity, health status and mental distress in individuals with and without cancer according to comorbidity type. We also examined the patterns of multimorbidity by identifying clusters of conditions and their associations with health outcomes.

## 2 METHODS

### 2.1 Data source

We performed a cross‐sectional analysis using data from the Australian National Health Survey 2017–2018. The survey population was randomly selected using a stratified multistage area approach and covered a representative sample of 21,315 individuals residing in private dwellings in urban and rural areas from across all Australian states and territories (response rate: 76.1% and the details have been described elsewhere).^9^ The survey excluded people living in non‐private dwellings such as hospitals, hotels, nursing homes and short‐stay caravan parks, as well as people residing in very remote areas of Australia and discrete communities. The trained Australian Bureau of Statistics interviewers conducted personal interviews with the selected sample population and collected a range of health‐related information including long‐term health conditions, health status and health risk factors. We obtained basic unit record information including demographic, and health characteristics for each person that participated in the survey.^10^

Study population consisted of all survey respondents aged ≥25 years (selected based on the survey's predefined age groups) and included individuals with and without a history of cancer (skin malignant neoplasms and/or malignant neoplasms of other sites or site unknown). We categorised the study population into two groups (cancer versus non‐cancer) and included all cancer cases (whether cancer is current or in remission) to reflect the characteristics of cancer survivorship. Of note, the information on types and stages of cancer, as well as the date of cancer diagnosis was not available.

### 2.2 Comorbidity

The survey collected health‐related data including information on common health conditions. Participants were asked if they have any long‐term health conditions that have lasted or are expected to last, for 6 months or more. These health conditions were coded using the International Classification of Diseases 10th revision (ICD‐10) and were categorised into broad disease grouping including diseases of circulatory, respiratory, nervous, digestive, genitourinary, musculoskeletal and endocrine systems, disorders of blood, skin, eye, mental and behavioural problems and certain infections (Table S1). We identified all the coexisting health conditions reported by the study population that were both ‘current’ and long‐term. If a person reported having multiple health conditions that fell in the same broad disease grouping, we counted that disease grouping once only. A total of 12 broad disease groupings (‘comorbidity’) were included in our analysis.

### 2.3 Outcome measures

The outcome measures were self‐reported health status and mental distress.

Health status measured a person's perception of health at a given time point, where the study population was asked ‘In general would you say that your health is excellent, very good, good, fair or poor?’. This single‐item self‐reported scale is commonly used to provide a broad picture of a population's overall health^11^ and has also been shown to be a predictor of mortality.^12^, ^13^, ^14^ We grouped the measurement of health status into two categories (poor health status with a rating of fair or poor vs. good health status with a rating of excellent, very good or good).^1^

Mental distress was measured by the Kessler Psychological Distress Scale‐10 (K10) which assessed a person's level of psychological fatigue, nervousness, agitation and depression in the last 4 weeks.^15^ The 10‐item questionnaire used a five‐level response scale to each question that were scored from five through to one with a maximum total score of 50 (indicating severe distress) and a minimum total score of 10 (indicating no distress).^15^, ^16^ The validity of the K10 as a measure of psychological distress has been demonstrated in previous study based on the data of sensitivity and specificity presented.^16^ We grouped the level of mental distress into two categories (low‐moderate level of distress with a K10 score of 10–21 vs. high‐very high level of stress with a K10 score of 22–50).^1^, ^15^

### 2.4 Statistical analysis

Descriptive statistics were used to compare the demographic and characteristics of subjects based on their cancer status. The self‐reported outcomes of poor health status and mental distress were assessed using separate multivariate logistic regression models with each specific broad disease grouping as the exposure variable and adjustment for sociodemographic factors including sex, age, country of birth, highest education level, geographical location, and socioeconomic status by household income, and lifestyle factors including smoker status, dietary intake, alcohol consumption, physical activity and body mass index. The number of other broad disease groupings (i.e. conditions other than the specific disease grouping of interest) was also included each model. Results were reported as adjusted odds ratio (aOR) with 95% confidence intervals (CIs).

We also performed a k‐modes cluster analysis, to explore patterns of multimorbidity (i.e. two or more chronic health conditions excluding cancer) by cancer status.^17^ The optimal number of clusters was determined using the modified Elbow method, which suggested 4 to 6 clusters. The 4‐cluster solution was selected based on parsimony and alignment with clinical experience. Based on the prevalence of broad disease groupings within each cluster, we subjectively labelled the four clusters according to the most dominant conditions.

We assigned weighting to the study population by using the 2016 Census Australian population as the standard population (by 5‐year age groups and sex). The weight was applied in all analyses. Analysis was conducted using SAS statistical software version 9.4 and Python version 3.9.13 for the K‐modes clustering.

## 3 RESULTS

### 3.1 Cohort characteristics

There were 1982 individuals (52% female) with cancer and 12,635 (51% female) without cancer (Table 1). Individuals with cancer were older (53% vs. 18% aged ≥65 years), more likely to be born in Australia (80% vs. 67%), reside in inner regional areas (24% vs. 19%), and have a lower education level (42% vs. 33% had no non‐school qualification) and a lower socioeconomic status (25% vs. 16% in the lowest household income level) compared with those without cancer. They were more likely to be overweight or obese (74% vs. 69%), be current or ex‐smoker (56% vs. 49%), and physically inactive (23% vs. 16%) compared with those without cancer.

**TABLE 1:** Cohort characteristics.

Characteristics	Population weighted, *n* (%)	
	Cancer, total *N* = 1982	Non‐cancer, total *N* = 12,635
Sex		
Male	957 (48)	6130 (48)
Female	1025 (52)	6505 (51)
Age group in years		
25–34	65 (3)	3005 (24)
35–49	241 (12)	4060 (32)
50–64	629 (32)	3266 (26)
≥ 65	1047 (53)	2304 (18)
Country of birth		
Australia	1578 (80)	8495 (67)
Main English‐speaking countries	235 (12)	1385 (11)
Others	169 (9)	2755 (22)
Geographical location		
Major cities	1110 (56)	7838 (62)
Inner regional	483 (24)	2443 (19)
Other	389 (20)	2354 (19)
Highest education attainment		
Postgraduate	167 (8)	1389 (11)
Bachelor degree	244 (12)	2547 (20)
Diploma	194 (10)	1413 (11)
Certificate	475 (24)	2741 (22)
No non‐school qualification	825 (42)	4125 (33)
Level not determined	77 (4)	420 (3)
Socioeconomic status (household income)		
Decile 1–2 (lowest)	495 (25)	1963 (16)
Decile 3–4	432 (22)	1947 (15)
Decile 5–6	330 (17)	2175 (17)
Decile 7–8	233 (12)	2433 (19)
Decile 9–10	281 (14)	2528 (20)
Missing	211 (11)	1589 (13)
Self‐reported body mass index		
Normal weight	483 (24)	3814 (30)
Underweight	21 (1)	125 (1)
Overweight	742 (37)	4555 (36)
Obese	736 (37)	4141 (33)
Smoker status		
Never smoked	878 (44)	6394 (51)
Ex‐smoker	817 (41)	4094 (32)
Current smoker	287 (15)	2147 (17)
Dietary intake: meet fruit and vegetables consumption guidelines		
Met both fruit and vegetable guidelines	141 (7)	683 (5)
Met vegetable guideline only	61 (3)	298 (2)
Met fruit guideline only	928 (47)	5714 (45)
Did not meet either guideline	852 (43)	5940 (47)
Alcohol consumption (last 12 months)		
Not applicable	456 (23)	2619 (21)
1–7 days a week	970 (49)	5857 (46)
1–3 days a month	267 (13)	2207 (17)
Less than once a month	284 (14)	1905 (15)
Not known	5 (<1)	47 (<1)
Physical activity (in the last week)		
High	211 (11)	2306 (18)
Moderate	593 (30)	4301 (34)
Low‐very low	716 (36)	3979 (31)
No physical activity	453 (23)	1988 (16)
Not stated	9 (<1)	61 (<1)

### 3.2 Comorbidity prevalence

Comorbidity was common in individuals with cancer (*n* = 1801, 91%) (Table 2). The most prevalent type of comorbidity reported in individuals with cancer was diseases of the musculoskeletal (63%), circulatory (43%), respiratory (38%), and endocrine systems (33%), and mental and behavioural problems (31%). The most prevalent type of comorbidity reported in individuals without cancer was diseases of the musculoskeletal (38%) and respiratory systems (34%), mental and behavioural problems (27%), and diseases of the circulatory (21%) and endocrine systems (18%).

**TABLE 2:** Prevalence of comorbidity and self‐report health outcomes by cancer status.

Characteristics	Population weighted, *n* (%)	
	Cancer, total *N* = 1982	Non‐cancer, total *N* = 12,635
Number of chronic conditions (excluding cancer)		
0	181 (9)	3101 (25)
1–2	811 (41)	6172 (49)
3–4	702 (35)	2643 (21)
≥ 5	288 (15)	719 (6)
Number of chronic conditions (excluding cancer)		
Median (Q1, Q3)	2 (1, 4)	1 (1, 3)
Mean (SD)	2.66 (1.62)	1.72 (1.52)
Comorbidities		
Diseases of musculoskeletal system and connective tissue		
Yes	1253 (63)	4833 (38)
No	729 (37)	7802 (62)
Diseases of circulatory system		
Yes	855 (43)	2693 (21)
No	1127 (57)	9942 (79)
Diseases of respiratory system		
Yes	762 (38)	4307 (34)
No	1220 (62)	8328 (66)
Endocrine, nutritional and metabolic diseases		
Yes	648 (33)	2259 (18)
No	1334 (67)	10,376 (82)
Mental and behavioural problems		
Yes	609 (31)	3401 (27)
No	1373 (69)	9234 (73)
Diseases of digestive system		
Yes	299 (15)	964 (8)
No	1683 (85)	11,671 (92)
Diseases of eye and adnexa		
Yes	250 (13)	565 (4)
No	1732 (87)	12,070 (96)
Diseases of nervous system		
Yes	215 (11)	1201 (10)
No	1767 (89)	11,434 (90)
Diseases of genitourinary system		
Yes	203 (10)	627 (5)
No	1779 (90)	12,008 (95)
Diseases of skin and subcutaneous tissue		
Yes	97 (5)	546 (4)
No	1885 (95)	12,089 (96)
Diseases of blood and blood forming organs		
Yes	66 (3)	292 (2)
No	1916 (97)	12,343 (98)
Certain infectious and parasitic diseases		
Yes	11 (1)	71 (1)
No	1971 (99)	12,564 (99)
Self‐reported health status		
Good	1438 (73)	10,672 (84)
Poor	544 (27)	1963 (16)
Mental distress (K10)		
Low‐moderate distress level	1633 (82)	10,584 (84)
High‐very high distress level	290 (15)	1662 (13)
Not asked/unable to determine	59 (3)	389 (3)

### 3.3 Comorbidities and odds of poor health/mental distress

Amongst individuals with cancer and a comorbidity, the odds of reporting either poor health or mental distress was more than six times higher than those without cancer and without a comorbidity (Table 3). Amongst the specific broad diseases, individuals with cancer and each specific broad disease were more likely to report poor health than those without cancer for each broad disease grouping except for skin conditions and infectious diseases. The aOR ranged from 1.34 (95% CI = 1.01–1.79) for digestive disorders to 2.93 (95% CI = 1.62–5.29) for blood conditions.

**TABLE 3:** Adjusted odds ratios for poor health status and mental distress by comorbidity status.

Comorbidity status by broad disease groupings	Adjusted odds ratios^a^ (95% confidence interval)	
	Self‐reported health status	Mental distress
	Poor health versus good health (reference)	High distress versus low distress (reference)
Any comorbidity		
Non‐cancer without any comorbidity	Reference	Reference
Non‐cancer with a comorbidity	4.92 (4.01–6.03)	5.92 (4.78–7.32)
Cancer without any comorbidity	1.03 (0.47–2.24)	0.41 (0.09–1.96)
Cancer with a comorbidity	6.44 (5.09–8.14)	6.75 (5.22–8.72)
Mental and behavioural problems		
Non‐cancer without mental problems	Reference	Reference
Non‐cancer with mental problems	2.69 (2.39–3.02)	9.56 (8.40–10.88)
Cancer without mental problems	1.31 (1.10–1.55)	1.30 (1.00–1.69)
Cancer with mental problems	2.79 (2.27–3.43)	9.01 (7.25–11.20)
Diseases of musculoskeletal system and connective tissue (MSK)		
Non‐cancer without MSK disease	Reference	Reference
Non‐cancer with MSK disease	1.84 (1.64–2.07)	1.51 (1.33–1.71)
Cancer without MSK disease	1.21 (0.94–1.54)	1.03 (0.77–1.38)
Cancer with MSK disease	2.11 (1.78–2.52)	1.43 (1.16–1.75)
Diseases of nervous system (NS)		
Non‐cancer without NS disease	Reference	Reference
Non‐cancer with NS disease	1.77 (1.50–2.08)	1.56 (1.33–1.84)
Cancer without NS disease	1.20 (1.04–1.39)	0.94 (0.79–1.12)
Cancer with NS disease	1.68 (1.20–2.34)	1.67 (1.18–2.36)
Diseases of blood and blood forming organs		
Non‐cancer without blood disease	Reference	Reference
Non‐cancer with blood disease	1.92 (1.41–2.61)	1.49 (1.09–2.02)
Cancer without blood disease	1.16 (1.01–1.33)	0.97 (0.82–1.14)
Cancer with blood disease	2.93 (1.62–5.29)	1.27 (0.68–2.35)
Diseases of genitourinary system		
Non‐cancer without genitourinary disease	Reference	Reference
Non‐cancer with genitourinary disease	1.73 (1.41–2.13)	1.42 (1.13–1.77)
Cancer without genitourinary disease	1.18 (1.02–1.36)	1.00 (0.84–1.18)
Cancer with genitourinary disease	1.91 (1.37–2.67)	1.13 (0.76–1.65)
Diseases of circulatory system (CVD)		
Non‐cancer without CVD disease	Reference	Reference
Non‐cancer with CVD disease	1.65 (1.45–1.87)	1.12 (0.96–1.29)
Cancer without CVD disease	1.29 (1.07–1.55)	1.05 (0.85–1.30)
Cancer with CVD disease	1.74 (1.44–2.11)	0.98 (0.77–1.24)
Diseases of eye and adnexa		
Non‐cancer without eye disease	Reference	Reference
Non‐cancer with eye diseases	1.26 (1.01–1.57)	1.07 (0.82–1.39)
Cancer without eye disease	1.16 (1.00–1.34)	0.95 (0.80–1.12)
Cancer with eye disease	1.60 (1.18–2.18)	1.21 (0.83–1.77)
Endocrine, nutritional and metabolic diseases		
Non‐cancer without endocrine disease	Reference	Reference
Non‐cancer with endocrine disease	1.49 (1.31–1.70)	1.07 (0.92–1.25)
Cancer without endocrine disease	1.23 (1.04–1.46)	0.92 (0.75–1.12)
Cancer with endocrine disease	1.59 (1.29–1.95)	1.10 (0.86–1.41)
Diseases of respiratory system		
Non‐cancer without respiratory disease	Reference	Reference
Non‐cancer with respiratory disease	1.30 (1.16–1.46)	1.24 (1.10–1.39)
Cancer without respiratory disease	1.26 (1.06–1.49)	0.99 (0.79–1.22)
Cancer with respiratory disease	1.36 (1.11–1.65)	1.15 (0.91–1.44)
Diseases of digestive system		
Non‐cancer without digestive disease	Reference	Reference
Non‐cancer with digestive disease	1.42 (1.19–1.70)	1.29 (1.06–1.55)
Cancer without digestive disease	1.23 (1.06–1.42)	1.04 (0.88–1.24)
Cancer with digestive disease	1.34 (1.01–1.79)	0.91 (0.65–1.28)
Diseases of skin and subcutaneous tissue		
Non‐cancer without skin disease	Reference	Reference
Non‐cancer with skin disease	1.09 (0.85–1.40)	1.14 (0.89–1.47)
Cancer without skin disease	1.16 (1.01–1.33)	0.97 (0.82–1.14)
Cancer with skin disease	1.43 (0.87–2.34)	0.92 (0.51–1.66)
Certain infectious and parasitic diseases		
Non‐cancer without infections	Reference	Reference
Non‐cancer with infections	3.19 (1.81–5.63)	1.41 (0.75–2.66)
Cancer without infections	1.18 (1.03–1.35)	0.96 (0.82–1.13)
Cancer with infections	1.26 (0.30–5.22)	1.40 (0.29–6.80)

^a^ The logistic regression model was adjusted for sociodemographic factors including sex, age, country of birth, highest education level, geographical location and socioeconomic status by household income and lifestyle factors including smoker status, dietary intake (whether meet fruit and vegetables consumption guidelines), alcohol consumption in the last 12 months, physical activity in the past week and body mass index. For the analysis by individual type of broad disease groupings, the number of other broad disease groupings (i.e. conditions other than the health condition of interest) was also included in the model.

A comorbid mental illness was associated with the highest odds of reporting both poor health (aOR 2.79, 95% CI = 2.27–3.43) and mental distress (aOR 9.01, 95% CI = 7.25–11.20) in individuals with cancer. This was followed by comorbid musculoskeletal and nervous system disorders (aOR of reporting poor health status ranged from 1.68 to 2.11 and aOR of reporting mental distress ranged from 1.43 to 1.67).

A comorbid blood condition was associated with an increased odds of reporting poor health (aOR 2.93, 95% CI = 1.62–5.29) but not distress (aOR 1.27, 95% CI = 0.68–2.35) in individuals with cancer. Similar patterns were observed for comorbid genitourinary, circulatory, eye, endocrine, respiratory and digestive systems (in order of decreasing magnitude odds of reporting poor health status).

### 3.4 Cancer and non‐cancer clusters

About 70% (*n* = 1418) of individuals with cancer and nearly 50% (*n* = 6081) of those without cancer had multimorbidity (≥2 comorbidities). Four distinct and similar patterns of clustering were observed in both the cancer and non‐cancer groups (Table 4). Cluster 1 (low comorbidity) represented 32% of those with cancer and 34% of those without cancer and was characterised by a relatively low prevalence of each disease grouping ranging from 0% to 28% in the cancer group and 0–9% in the non‐cancer group. Cluster 2 (musculoskeletal) had a predominance of musculoskeletal conditions (100% of individuals with cancer and 73% of individuals without cancer) and a high prevalence of mental illnesses (69% of individuals without cancer). Cluster 3 (respiratory) had high prevalence of respiratory conditions (100% of individuals with and without cancer) in addition to 100% with musculoskeletal disease amongst those with cancer. Cluster 4 (cardiometabolic) represented 21% of those with cancer and 12% of those without cancer and included a high prevalence of diseases of the circulatory and endocrine systems amongst both the cancer (100% for both conditions) and non‐cancer (88% circulatory and 77% endocrine) populations. The prevalence of mental disorders was evenly distributed across the four clusters in the cancer groups, while over two‐thirds of individuals without cancer and in cluster 2 had a mental disorder.

**TABLE 4:** The characteristics of study population by cancer status and cluster of multimorbidity.

Characteristics	Population weighted, *n* (%)							
	Cancer, Total *N* = 1982				Non‐cancer, Total *N* = 12,635			
	Cluster 1, *n* = 627 (32%): *low comorbidity cluster*	Cluster 2, *n* = 521 (26%): *MSK cluster*	Cluster 3, *n* = 409 (21%): *respiratory cluster*	Cluster 4, *n* = 425 (21%): *cardio*‐*metabolic cluster*	Cluster 1, *n* = 4325 (34%): *low comorbidity cluster*	Cluster 2, *n* = 3524 (28%): *MSK cluster*	Cluster 3, *n* = 3235 (26%): *respiratory cluster*	Cluster 4, *n* = 1551 (12%): *cardio*‐*metabolic cluster*
Sex								
Female	288 (46)	279 (54)	241 (59)	217 (51)	2017 (47)	1923 (55)	1705 (53)	860 (55)
Age group in years								
25–34	43 (7)	10 (2)	11 (3)	1 (<1)	1366 (32)	753 (21)	840 (26)	46 (3)
35–49	140 (22)	44 (8)	46 (11)	11 (3)	1558 (36)	1201 (34)	1137 (35)	164 (11)
50–64	224 (36)	171 (33)	134 (33)	100 (23)	938 (22)	997 (28)	789 (24)	542 (35)
≥ 65	220 (35)	296 (57)	218 (53)	313 (74)	463 (11)	573 (16)	469 (14)	799 (52)
Country of birth								
Australia	501 (80)	411 (79)	333 (81)	333 (78)	2637 (61)	2563 (73)	2263 (70)	1032 (67)
Main English‐speaking countries	79 (13)	63 (12)	39 (10)	54 (13)	453 (10)	379 (11)	349 (11)	204 (13)
Others	47 (7)	47 (9)	37 (9)	38 (9)	1235 (29)	582 (17)	623 (19)	315 (20)
Highest education attainment								
Postgraduate	65 (10)	41 (8)	30 (7)	31 (7)	560 (13)	346 (10)	357 (11)	126 (8)
Bachelor degree	96 (15)	59 (11)	47 (11)	42 (10)	1025 (24)	611 (17)	752 (23)	159 (10)
Diploma	70 (11)	43 (8)	36 (9)	45 (11)	464 (11)	428 (12)	394 (12)	127 (8)
Certificate	164 (26)	111 (21)	114 (28)	86 (20)	900 (21)	797 (23)	718 (22)	326 (21)
No non‐school qualification	215 (34)	245 (47)	163 (40)	202 (48)	1232 (28)	1248 (35)	887 (27)	758 (49)
Level not determined	17 (3)	22 (4)	19 (5)	19 (4)	144 (3)	94 (3)	127 (4)	55 (4)
Geographical location								
Major cities	368 (59)	283 (54)	224 (55)	235 (55)	2702 (62)	2120 (60)	2113 (65)	903 (58)
Inner regional	131 (21)	130 (25)	112 (27)	110 (26)	727 (17)	728 (21)	610 (19)	378 (24)
Other	128 (20)	108 (21)	73 (18)	80 (19)	896 (21)	676 (19)	512 (16)	270 (17)
Socioeconomic status (household income)								
Decile 1–2 (lowest)	113 (18)	127 (24)	118 (29)	137 (32)	466 (11)	670 (19)	433 (13)	394 (25)
Decile 3–4	105 (17)	118 (23)	90 (22)	119 (28)	547 (13)	614 (17)	429 (13)	357 (23)
Decile 5–6	106 (17)	87 (17)	72 (18)	65 (15)	779 (18)	606 (17)	561 (17)	229 (15)
Decile 7–8	100 (16)	61 (12)	36 (9)	36 (8)	916 (21)	621 (18)	712 (22)	184 (12)
Decile 9–10	136 (22)	72 (14)	49 (12)	24 (6)	1011 (23)	596 (17)	735 (23)	186 (12)
Missing	67 (11)	56 (11)	44 (11)	44 (10)	606 (14)	417 (12)	365 (11)	201 (13)
Number of chronic conditions (excl cancer)								
Median (Q1, Q3)	1 (0, 2)	2 (1, 3)	4 (3, 4)	4 (3, 5)	0 (0, 1)	2 (1, 3)	2 (1, 3)	3 (2, 4)
Mean (SD)	1.19 (1.02)	2.27 (1.01)	3.64 (1.10)	4.37 (1.38)	0.33 (0.58)	2.29 (1.38)	2.11 (1.03)	3.48 (1.36)
Broad disease groupings								
Diseases of musculoskeletal system and connective tissue	0 (0)	521 (100)	409 (100)	323 (76)	0 (0)	2576 (73)	976 (30)	1281 (83)
Diseases of circulatory system	124 (20)	166 (32)	140 (34)	425 (100)	375 (9)	424 (12)	529 (16)	1365 (88)
Diseases of respiratory system	174 (28)	0 (0)	409 (100)	179 (42)	0 (0)	734 (21)	3235 (100)	338 (22)
Endocrine, nutritional and metabolic diseases	83 (13)	75 (14)	65 (16)	425 (100)	313 (7)	335 (10)	416 (13)	1195 (77)
Mental and behavioural problems	148 (24)	154 (30)	156 (38)	151 (35)	0 (0)	2438 (69)	579 (18)	384 (25)
Diseases of digestive system	59 (9)	68 (13)	81 (20)	91 (21)	158 (4)	353 (10)	253 (8)	200 (13)
Diseases of eye and adnexa	45 (7)	59 (11)	56 (14)	90 (21)	94 (2)	164 (5)	113 (3)	194 (13)
Diseases of nervous system	47 (7)	41 (8)	79 (19)	48 (11)	214 (5)	497 (14)	359 (11)	131 (8)
Diseases of genitourinary system	23 (4)	53 (10)	46 (11)	81 (19)	105 (2)	218 (6)	130 (4)	174 (11)
Diseases of skin and subcutaneous tissue	26 (4)	24 (5)	25 (6)	22 (5)	107 (2)	197 (6)	157 (5)	85 (5)
Diseases of blood and blood forming organs	11 (2)	18 (3)	17 (4)	20 (5)	53 (1)	121 (3)	75 (2)	43 (3)
Certain infectious and parasitic diseases	3 (<1)	2 (<1)	3 (1)	3 (1)	20 (<1)	26 (1)	16 (1)	9 (1)

Abbreviation: MSK, musculoskeletal.

**FIGURE 1:**
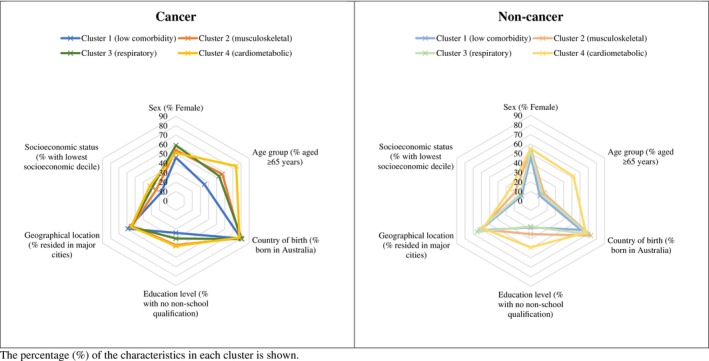
The prevalence distribution of the study population characteristics by cancer status and clusters of multimorbidity. The percentage (%) of the characteristics in each cluster is shown.

Across the four clusters, for both those with and for those without cancer, those in cluster 4 (cardiometabolic cluster) were older compared with those in clusters 1–3 (Table 4, Figure 1). Individuals in cluster 4 were also more likely to have a lower education level and a lower socioeconomic status compared with those in other clusters. In contrast, individuals in cluster 1 (low comorbidity) were younger, more likely to have a postgraduate/bachelor's degree qualification and a higher socioeconomic status compared with those in clusters 2–4.

**FIGURE 2:**
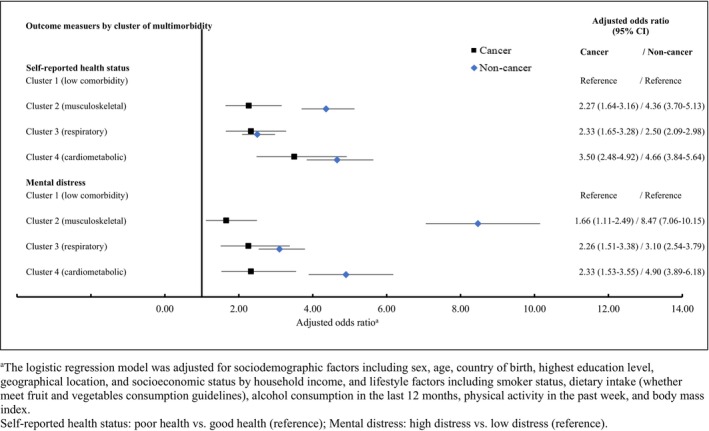
Adjusted odds ratios for poor health status and mental distress by cancer status and by cluster of multimorbidity. ^a^The logistic regression model was adjusted for sociodemographic factors including sex, age, country of birth, highest education level, geographical location, and socioeconomic status by household income, and lifestyle factors including smoker status, dietary intake (whether meet fruit and vegetables consumption guidelines), alcohol consumption in the last 12 months, physical activity in the past week and body mass index. Self‐reported health status: poor health versus good health (reference); mental distress: high distress versus low distress (reference).

When assessing the odds of poor health and mental distress broken down by cancer status and cluster, compared to cluster 1 (low comorbidity), those in clusters 2–4 were more likely to report poor health amongst those with cancer (aORs: 2.27–3.50) and those without cancer (aORs: 2.50–4.66) (Figure 2). Individuals in clusters 2–4 also had higher odds of mental distress than those in cluster 1 for both those with cancer (aORs: 1.66–2.33) and for those without cancer (aORs: 3.10–8.47). Cardiometabolic disease (cluster 2) was associated with the highest odds of reporting both poor health (aOR 3.50, 95% CI = 2.48–4.92) and mental distress (aOR 2.33, 95% CI = 1.53–3.55) amongst individuals with cancer.

## 4 DISCUSSION

In this first Australian study that assessed the effects of comorbid conditions on health outcomes in individuals with cancer, we found that the presence of comorbidity was more prevalent and associated with a poor health status in cancer survivors as compared to individuals without cancer; and the magnitude of the association varied by comorbidity type. A comorbid mental illness was associated with the worst self‐reported health status and mental distress in cancer survivors. Our study provides an important overview on the patterns of health outcomes in the context of different comorbidities.

The worst health outcomes (e.g. a higher odds of mental distress as measured by the K10 score as an indicator for the needs of mental health services)^16^ amongst cancer survivors with comorbid mental illness observed in our study are consistent with prior research which showed that mental condition after cancer had negative impacts on physical morbidity and mortality.^18^, ^19^ Previous studies showed that while the incidence of cancer was similar in people with psychiatric disorders to that in the general population, those with mental illness were more likely to have metastases at cancer diagnosis and were less likely to receive cancer treatments which may contribute to their poorer health outcome.^20^, ^21^ Cancer diagnosis, and the resulting fear of cancer recurrence can lead to psychological distress and the development of new mental health issues such as depression and anxiety which can adversely impact health status.^22^, ^23^ Our findings of worst outcomes in cancer survivors with comorbid mental illness are particularly significant as the psychosocial needs for cancer survivors remain frequently unmet.^18^

Musculoskeletal disorder was the most common comorbidity reported amongst cancer survivors in our study and was also associated with poor health status and mental distress. Musculoskeletal disorders are the leading contributor to disability and chronic pain in Australia.^24^ A higher prevalence of musculoskeletal conditions in people with cancer may be due to cancer treatment such as hormonal therapy‐induced osteoporosis and arthralgia in people with breast or prostate cancer, the most prevalent cancers in Australia.^25^, ^26^ Musculoskeletal symptoms are known to contribute to hormonal therapy discontinuation which may have negative implications on cancer outcomes.^26^ In addition to cancer, the functional limitations, pain and distress caused by musculoskeletal conditions can have deleterious effect on an individual's ability to stay mobile and altered quality of life, leading to physical and social decline.^27^ Our findings highlight the importance of the assessment and management of musculoskeletal conditions as part of the standard practice in cancer care.

Although there were no significant differences in mental distress in individuals with cancer and comorbid conditions including diseases of blood, genitourinary, circulatory, eye, endocrine, respiratory and digestive systems as compared to individuals without cancer and without the specific comorbidity type, the presence of those illnesses increased the odds of reporting poor health. Cardiovascular conditions, deserve particular attention as they affect a significant proportion of people with cancer and are the leading cause of premature non‐cancer death amongst long‐term cancer survivors, underscoring the importance of their proactive assessment and management.^28^, ^29^

Our exploratory analysis of patterns of multimorbidity showed that 70% of individuals with cancer had multimorbidity with four distinct patterns of clustering observed, which was also observed in individuals without cancer. A high prevalence of cardiometabolic diseases in cancer group (cluster 4) may be the reflection of the risk factors shared between cancer and cardiometabolic diseases or cardiovascular toxicity associated with cancer treatment or a reflection of the older age of individuals in the cancer cohort.^1^, ^2^ Most cancer cases occur in older age groups whereby the coexistence of multiple chronic diseases is common as the incidence of chronic diseases increases with age.^2^ Our results were consistent with prior studies which showed that cardiovascular diseases was associated with poor health‐related quality of life in the general population.^30^ Further, the clusters with a higher number of comorbidity (clusters 2–4) comprised a higher proportion of individuals with a lower socioeconomic status when compared to those with a low comorbidity (cluster 1) in both the cancer and non‐cancer groups, consistent with what is observed in people with multimorbidity in the general population.^31^ A systematic review included 24 cross‐sectional studies that examined the association between socioeconomic status and the occurrence of multimorbidity showed that a lower versus higher socioeconomic status (as measured by education level and area‐based deprivation) was generally linked to an increased risk of multimorbidity.^31^

Our findings suggests that the presence of multimorbidity, particularly in those with cardiometabolic diseases and socioeconomic disparity should alert clinicians to a higher risk for inferior outcomes. Our results highlight the importance of the management of multiple chronic diseases as outlined in the ‘National Strategic Framework for Chronic Conditions’ including integrating primary care into the model of care for cancer survivors.^32^ Future research into the development of cluster‐specific care management may also be of value.^33^

Our research has several limitations. While we were able to adjust for several important characteristics including sex, age, sociodemographic such as socioeconomic status by household income and education level, lifestyle factors and presence of other health conditions, the health data used in this study was self‐reported which may be subject to response bias. The information was not available in the dataset including cancer stage/type and treatment as well as the dates when cancer and other health conditions were diagnosed. Therefore, we were not able to perform further subgroup analysis by types of cancer. We were also not able to differentiate the sequences of comorbidities development and health outcomes in relation to cancer diagnosis. Nonetheless, our study provided an important overview on the effects of individual comorbid conditions on health outcomes given that there is limited data available in the Australian context.

In conclusion, comorbidities in people with cancer are common and associated with inferior health status. The strongest association with poor health and mental distress was observed for those with a mental illness and those in the cluster with a high prevalence of cardiometabolic diseases. Risk assessment and management of comorbidities should be an important priority for cancer care and research.

## AUTHOR CONTRIBUTIONS

**Huah Shin Ng:** Conceptualization (lead); data curation (lead); formal analysis (lead); investigation (lead); methodology (lead); project administration (lead); visualization (lead); writing – original draft (lead); writing – review and editing (lead). **Richard Woodman:** Conceptualization (supporting); investigation (supporting); methodology (supporting); writing – review and editing (supporting). **Bogda Koczwara:** Conceptualization (supporting); investigation (supporting); methodology (supporting); writing – original draft (supporting); writing – review and editing (supporting).

## FUNDING INFORMATION

This research did not receive any specific grant from funding agencies.

## CONFLICT OF INTEREST STATEMENT

The authors have no conflicts of interest to disclose.

## ETHICS STATEMENT

Ethics approval was not required for the analysis of de‐identified National Health Survey basic microdata.

## Supporting information


Table S1.

## Data Availability

As we are not the data custodians, we are not authorised to make the data available. With the appropriate approvals, the data may be accessed through the Australian Bureau of Statistics.
