# Supplementary material for: Health status and mental distress in people with cancer and comorbid conditions: The Australian National Health Survey analysis
Source: Cancer Med. 2023 Jun 23;12(16):17225–38. doi: 10.1002/cam4.6291 (PMC10501298; doi:10.1002/cam4.6291)
Supplement: Supplementary file 1 — Table S1. [file CAM4-12-17225-s001.docx]

**Supplementary Table 1** Type of health conditions included in each broad disease grouping

| **Broad disease groupings** | **Type of conditions** |
| --- | --- |
| (1) Blood and blood forming organs | Anaemias, other diseases of blood and blood forming organs |
| (2) Endocrine, nutritional and metabolic diseases | Disorders of thyroid gland, diabetes mellitus type 1, diabetes mellitus type 2, diabetes mellitus type unknown, high sugar levels in blood/ urine, high cholesterol, gestational diabetes, other endocrine nutritional metabolic diseases |
| (3) Mental and behavioural problems | Alcohol and drug problems, feeling anxious/nervous/tense, feeling depressed, behavioural, cognitive and emotional problems with usual onset in childhood/adolescence, other mental and behavioural problems, other problems of psychological development, anxiety disorders, panic disorders/panic attacks, phobic anxiety disorders, post-traumatic stress disorder, other mood (affective disorders), autism spectrum disorders, depression, obsessive compulsive disorder |
| (4) Nervous system | Epilepsy, migraine, other diseases of nervous system |
| (5) Eye and adnexa (selected) | Macular degeneration, cataract, glaucoma, other disorders of choroid and retina |
| (6) Circulatory system | Other diseases of veins lymphatic vessels, diseases of arteries, arterioles & capillaries, low blood pressure, angina, oedema, other signs & symptoms involving circulatory system, tachycardia, other diseases of circulatory system, other heart diseases, other ischemic heart diseases, heart attack, heart failure, cardiac murmurs and cardiac sounds, hypertensive disease, other cerebrovascular disease, stroke (including after effects of stroke), varicose veins, haemorrhoids |
| (7) Respiratory system | Hay fever and allergic rhinitis, chronic sinusitis, bronchitis, emphysema, asthma, symptoms &signs involving respiratory system, all other diseases of respiratory system |
| (8) Digestive system | Other diseases of the intestines, other diseases of the oesophagus, stomach & duodenum, all other diseases of digestive system, diseases of oesophagus, stomach/duodenal/gastrointestinal ulcer, hernia, gallstones, symptoms& signs involving digestive system |
| (9) Skin and subcutaneous tissue | Dermatitis & eczema, psoriasis, symptoms & signs involving skin and subcutaneous tissue, other diseases of skin and subcutaneous tissue |
| (10) Musculoskeletal system and connective tissue | Other arthropathies, other soft tissue disorders, disc disorders, rheumatism, back pain/ problems not elsewhere classified, arthritis- osteoarthritis, arthritis- other and type unknown, curvature of the spine, sciatica, arthritis- rheumatoid, osteoporosis, gout, symptoms & signs involving nervous and musculoskeletal system, other diseases musculoskeletal system and connective tissue |
| (11) Genitourinary system | Other diseases of genitourinary system, incontinence: urine, kidney disease, urinary calculus, diseases of female pelvic organs and genital tract, diseases of male genital organs |
| (12) Certain infectious and parasitic diseases | Certain infectious and parasitic diseases |
